# Supplementary figures and images for: Screening microalgae isolated from urban storm- and wastewater systems as feedstock for biofuel
Source: PeerJ. 2016 Sep 1;4:e2396. doi: 10.7717/peerj.2396 (PMC5012288; doi:10.7717/peerj.2396)

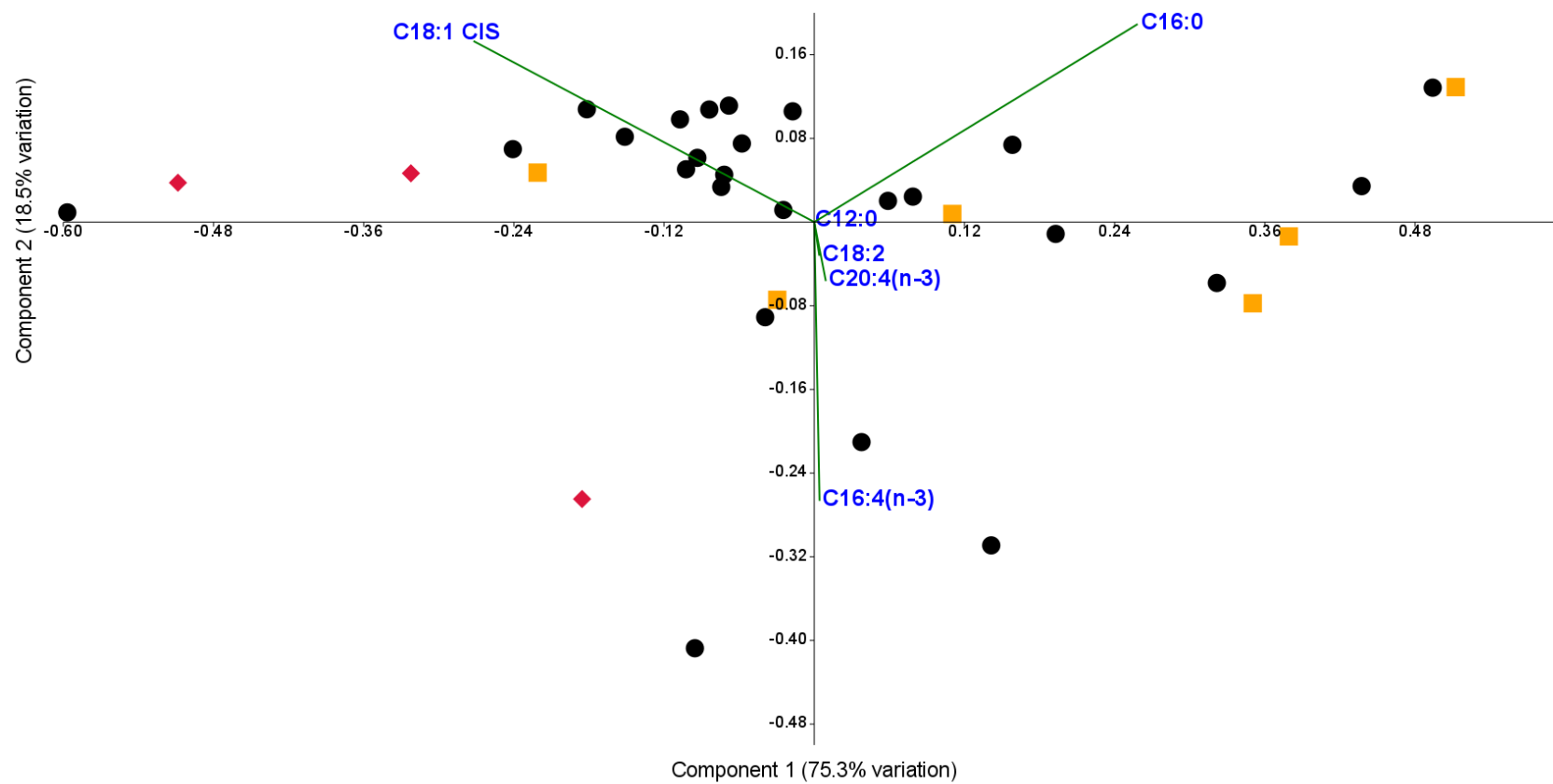

**Fig. 3**

Supplement: Supplemental Information 4 — Principal component analyses of fatty acid profiles for thirty-four algal strains labelled by source location: Black circle = Stormwater Pond, Orange square = natural reference site, Red Diamond = Municipal Wastewater Treatment Plant. Vector lines represent individual fatty acids. [file peerj-04-2396-s004.pdf]
